# Biomarkers in Coronary Artery Bypass Surgery: Ready for Prime Time and Outcome Prediction?

**DOI:** 10.3389/fcvm.2015.00039

**Published:** 2016-01-05

**Authors:** Alessandro Parolari, Paolo Poggio, Veronika Myasoedova, Paola Songia, Giorgia Bonalumi, Alberto Pilozzi, Davide Pacini, Francesco Alamanni, Elena Tremoli

**Affiliations:** ^1^Dipartimento di Scienze Biomediche per la Salute, Università Degli Studi di Milano, Milan, Italy; ^2^Unità Operativa di Cardiochirurgia e Ricerca Traslazionale, San Donato IRCCS, San Donato Milanese, Milan, Italy; ^3^Centro Cardiologico Monzino IRCCS, Milan, Italy; ^4^Dipartimento di Scienze Farmacologiche e Biomolecolari, Università Degli Studi di Milano, Milan, Italy; ^5^Sezione Cardiovascolare, Dipartimento di Scienze Cliniche e di Comunità, Università Degli Studi di Milano, Milan, Italy; ^6^S.Orsola-Malpighi, Dipartimento di Cardiochirurgia, Università di Bologna, Bologna, Italy

**Keywords:** coronary artery bypass, biomarkers, genetic, dynamic, outcome

## Abstract

Coronary artery bypass surgery (CABG) is still one of the most frequently performed surgical procedures all over the world. The results of this procedure have been constantly improved over the years with low perioperative mortality rates, with relatively low complication rates. To further improve these outstanding results, the clinicians focused their attention at biomarkers as outcome predictors. Although biological testing for disease prediction has already been discussed many times, the role of biomarkers in outcome prediction after CABG is still controversial. In this article, we reviewed the current knowledge regarding the role of genetic and dynamic biomarkers and their possible association with the occurrence of adverse clinical outcomes after CABG. We also took into consideration that the molecular pathway activation and the possible imbalance may affect hard outcomes and graft patency. We analyzed biomarkers classified in two different categories depending on their possibility to change over time: genetic markers and dynamic markers. Moreover, we evaluated these markers by dividing them, into sub-categories, such as inflammation, hemostasis, renin–angiotensin, endothelial function, and other pathways. We showed that biomarkers might be associated with unfavorable outcomes after surgery, and in some cases improved outcome prediction. However, the identification of a specific panel of biomarkers or of some algorithms including biomarkers is still in an early developmental phase. Finally, larger studies are needed to analyze broad panel of biomarkers with the specific aim to evaluate the prediction of hard outcomes and graft patency.

## Introduction

According to the European guidelines, coronary artery bypass surgery (CABG), which was introduced in the late sixties, associated with percutaneous intervention (PCI) is still one of the most frequently performed surgical procedures. CABG is still demonstrated to improve early-, mid-, and long-term results as regards event-free survival. Therefore, when patients are in elective surgery or the lesion is not treatable by PCI, CABG remains the treatment of choice not only in the case of left main trunk disease, reduced cardiac function or severe coronary disease (1, 2), but also in the case of diabetes (3, 4) and in the majority of patients affected by three-vessel disease (5, 6). Even if there have been complex clinical features of patients eligible for CABG, the early results of this procedure have been constantly improved over the years with low perioperative mortality rates, with relatively low complication rates [i.e., myocardial infarction (MI), stroke, and renal failure requiring dialysis (7–9)].

However, CABG gives rise to an important and sustained activation of different molecular pathways and cellular components. This leads to a persisting systemic inflammatory response associated with the activation of the hemostatic systems, with the concurrent occurrence of endothelial dysfunction and oxidative stress damage (10, 11).

It is well known that the incidence of adverse cardiovascular events, such as cardiac death, MI, stroke, graft occlusion and need for additional revascularization procedures, albeit relatively low in terms of numbers, can occur in the early days, weeks, and, subsequently, months after surgery (12–14). This could be explained by the activated molecular mechanisms in the perioperative period and in the early follow-up of CABG [i.e., pathways related to homeostasis and inflammation (15, 16)]. Moreover, a number of hereditary variables, in association with classic risk factors, might also be responsible for adverse events, suggesting a potential role of some gene polymorphisms.

In this article, we reviewed the current knowledge on molecular mechanisms and the association of biomarkers in the occurrence of adverse clinical outcomes after CABG, with a special emphasis on pathway activation and imbalance that may affect hard outcomes and graft patency.

## Biomarkers and Outcome Prediction?

Several translational studies have tried to address the question concerning the possible role of biological markers, and their underlying molecular mechanisms, in the prediction of major complications (graft patency included) after CABG.

Biomarkers should be divided into two different categories, depending on their possibility to change over time into (1) genetic markers, stable over time, and (2) dynamic markers, which may change mainly over time, but not only, perioperatively. In the next chapters, we examined the role of different biomarkers following this scheme.

## Genetic Markers

Gene polymorphisms and/or genetic mutations as new markers of morbidity and mortality in patients after CABG have been intensively studied over the past few years (17). Numerous studies in this field have been based on the identification of single nucleotide polymorphisms (SNPs) in candidate genes regulating inflammatory response (18, 19), thrombotic pathways (20, 21), oxidative stress (11), renin–angiotensin system (22, 23), cell damage (24, 25), and their association with post-operative results in patients undergoing CABG. The studies published so far and described in Tables S1–S5 in Supplementary Material based on specific pathways, have addressed two main questions: (1) can gene polymorphisms or mutations modulate or affect plasma levels of a dynamic biomarker, both at baseline or after the stimulus resulting from cardiac surgery? (2) can gene polymorphisms or mutations be associated with peri- and long post-operative outcomes?

### Inflammation

What emerges from currently available studies on inflammation is that gene polymorphisms may play a role in the modulation of some markers, such as IL-6 (18, 19, 26, 27), CRP (26–29), and TNF-α (30) (Table S1 in Supplementary Material). Moreover, they affect the levels of different biomarkers not only preoperative but also intra- and post-operative. In addition, there are other studies with limited sample size that need further evidence, suggesting a possible association between genetic polymorphisms and outcomes, such as the role of IL-6 (31) and CRP (31) genes in perioperative MI. Finally, the PEGASUS study, with a relatively large number of CABG patients, assessed the role of different categories of gene polymorphisms in association with the outcomes after CABG (20). This study showed that SNPs associated with inflammatory molecules [CRP, IL-1α, IL-1β, IL-6, IL-8, IL-10, interleukin 1 receptor antagonist (IL-1RN), and TNFα] did not affect long-term survival after CABG.

### Hemostasis

The same observation seems to be true for some of the hemostasis markers (Table S2 in Supplementary Material). However, we have reliable data regarding two markers: fibrinogen (23, 27), which is between inflammation and hemostasis; and beta-thromboglobulin (21). Interestingly, only one study has addressed the main issue of graft patency after CABG by assessing the presence of factor V Leiden genetic mutations (22). This study, limited by the small number of patients enrolled (a total of 100 CABGs), has suggested that the mutation Arg^506^Gln tends to be associated with saphenous graft occlusion [5/11 (45%) carriers vs. 18/89 (20%) non-carriers, *p* = 0.06] (32). Unfortunately, other studies on this subject analyzed only 2-year all causes mortality finding no associations (33), thus leaving the question on genetic bases of bypass patency open.

### Renin–Angiotensin

Regarding the renin–angiotensin system, most studies focused their attention on the gene coding for the angiotensin-converting enzyme (ACE; Table S3 in Supplementary Material). A study conducted by Welsby et al. (23) investigated and showed that ACE insertion/deletion (I/D) polymorphism plays a role in post-operative bleeding. On the contrary, the association of ACE I/D with cardiac mortality or all-cause mortality is controversial (22, 34). The large PEGASUS study showed no association with 5-year all-cause mortality (20), while a smaller study carried out by Völzke et al. (35) showed a strong association with both 2-year all-cause and cardiac mortality.

### Endothelial Function

There are other studies with limited sample size that need further evidence, suggesting a possible association between genetic polymorphisms and outcomes, such as the role of intercellular adhesion molecule-1 (ICAM-1) and of E-selectin 24 genes in perioperative MI (31, 36) (Table S4 in Supplementary Material). On the contrary, the PEGASUS study related to the endothelial function polymorphisms did not find any association between E-selectin, ICAM1, NOS3, PECAM1 VCAM1 SNPs, and 5-year all-cause mortality (20, 37).

### Other Pathways

Finally, there is also evidence suggesting that the post-operative levels of cell damage biomarker, cardiac Troponin-I, are affected by genetic variations. These occur in the chromosome 9 region p21 locus (24), suggesting a possible association between genetic profile and the risk of perioperative MI (Table S5 in Supplementary Material). This genetic variation (rs 10116277), which is adjacent or within genes involved in apoptosis, cell senescence, and cell cycle, significantly influences all-cause mortality at 5 years after CABG. This genetic variation has been demonstrated to substantially improve the predictive performance of the EuroSCORE risk algorithm (25). Moreover, together with two other variants found on the same chromosome region, it may also predict the occurrence of perioperative MI (24).

However, the studies reported in Tables S1–S5 in Supplementary Material have demonstrated and support the role of some genetic polymorphisms in the determination of plasma levels of some biomarkers. On the other hand, studies aim at demonstrating a possible relation between genetic variants and outcomes, such as mortality, stroke, or MI, failed so far because are seldom adequately powered to their scope. Thus, as regards current knowledge, there is not enough data to support or exclude such associations.

## Dynamic Markers

The behavior of what we call “dynamic” markers has been frequently assessed in the previous years in relation with perioperative outcomes. It is much easier to identify a marker represented by a continuous variable (e.g., CRP) as a potential independent predictor of a perioperative outcome itself compared to a categorical one (e.g., the presence of a genetic polymorphism). This is especially true in the case of low incidence rates. In the case of hard outcomes after CABG, the sample size to achieve a statistically significance may be excessively large. Moreover, since a number that can change over time represents dynamic markers, the correct timing for the assessment of the marker needs to be taken into consideration. In other words, not only the preoperative levels of the biomarker can be predictive of outcome but also the changes occurring perioperatively. The biomarkers much more frequently assessed in relation to outcome prediction belong mainly to inflammatory and hemostatic pathways.

### Inflammation

Inflammatory biomarkers have been frequently studied to assess their effect on early and long-term mortality, whereas hemostatic ones have been evaluated for their possible role in the occurrence of post-operative graft occlusion. As regards inflammatory pathways, several studies have shown that even non-specific inflammatory markers such as preoperative leukocyte count and CRP can predict in-hospital (38–41), mid- and long-term mortality after CABG (39, 42). Moreover, some small studies suggest that, besides mortality, increasing baseline CRP levels can also be an independent predictor both of early (43) and of late (44) major post-operative complications and that CRP levels may also play a role in early graft occlusion (45). In addition, IL-6 has been shown to predict the occurrence of early post-operative complications after surgery (19, 45, 46), such as early graft failure (45). Unfortunately, no information is currently available on the potential predictive power of the perioperative levels of both markers, and this could be of major interest as it is well known that they are both substantially unaffected by the use or the avoidance of the roller pump (47, 48). In this specific context, this feature could be important as these markers, in a near future, could become more reliable outcome predictors for CABG, whatever the technique chosen by the surgeon.

### Hemostatis

Concerning hemostasis, several different biomarkers have been assessed to predict perioperative bleeding and subsequent transfusion needs. Among them, preoperative fibrinogen (49, 50) and platelet aggregation impairment (51) have been associated with increased perioperative bleeding and transfusion risk. Interestingly, preoperative fibrinogen levels have a more predictive significance in women compared to men. It is known that women are at high risk for the need of perioperative blood transfusions (52) and previous interesting findings suggest that some degree of gender difference exists which may need further investigation (49). Other studies have also demonstrated that there might be a potential role for the changes of hemostatic biomarkers that occur perioperatively, thereby showing, once again, that perioperative levels of fibrinogen (up to 24 h after surgery) may affect bleeding.

Another important molecule involved in the hemostatic pathway is antithrombin III, a small protein that inactivates several enzymes of the coagulation system.

The levels of antithrombin III when measured upon Intensive Care Unit (ICU) arrival are predictive of several perioperative complications such as revision for bleeding, prolonged ICU and hospital stay, prolonged ventilation, neurological and thrombotic complications, blood transfusions (53, 54).

In our opinion, both fibrinogen and antithrombin levels are strictly associated with the consumption of the coagulation factors that occurs during surgery. As far as we know, the complexity of surgery and perfusion time changes this consumption, making it difficult to clarify the primary role of these two biomarkers. In other words, we consider it quite impossible to clarify the old concept of “who came first: the chicken or the egg?,” i.e., whether the *primum movens* is due to the decrease in levels of antithrombin itself or to the features and complexity of surgery that cause a decrease in antithrombin. Finally, some studies have also addressed the role of hemostatic perturbations and graft occlusion. These studies suggest that preoperative levels of thrombin generation markers (prothrombin fragment 1 + 2) (55) as well as fibrinolysis markers (tissue polypeptide antigen – tPA and antihemophilic factor VIII) (56) might be associated with reduced early graft patency. In addition, post-operative analysis (between 1 and 7 post-operative days) revealed the association between graft occlusion and increased activity of fibrinolytic marker PAI-1 (57, 58), increased levels of the thrombin generation marker (thrombin–antithrombin complexes) (56) and fibrinogen (56).

However, all these data should be taken with extreme caution as most of the studies were performed on a limited number of patients. The limited number of patients enrolled could be explained by their reluctance to undergo repeated coronary angiography after surgery, as most of these studies were performed before coronary computed tomography (CT) became available.

In the coming years, we expect that more studies will address the problem of graft patency after CABG, as coronary CT scan may favor patency assessment, thereby avoiding all the problems related to coronary angiography. In line with this latter statement, there is a novel study that was recently published from a sub-analysis of the radial patency study at 5-year follow-up (59). In this nested case–control sub-study, 87 patients were reassessed via coronary angiography or coronary CT scan at an average follow-up time of 8 ± 1.1 years after surgery. Twenty-six patients had an occluded radial or saphenous vein graft, whereas 61 did not. The analysis of fibrinogen levels showed that elevated levels of this molecule are associated with graft occlusion (59). However, the strength of this finding is sensibly weakened by the fact that blood collection was only done at the time of coronary angiography or CT scan, thus limiting the potential predictive role of fibrinogen itself.

All these data demonstrate that current knowledge about the role of biomarkers is still limited to the prognostic ability of some inflammation markers, mainly represented by CRP and to a lesser extent by IL-6. In conclusion, hemostatic markers have a definite role in perioperative bleeding and a much less definite role in hard outcomes and in graft patency.

## Conclusion

Current available data suggest that both genetic and dynamic markers may have a substantial correlation with outcomes. At present, it is too early to draw conclusions about the role of biomarkers on graft patency over time, but in the near future new technologies will be of great help.

What we know is that after coronary bypass surgery, there is a marked and protracted activation of several molecular pathways indicating increased inflammatory status, hemostasis activation, as well as increased oxidative stress and unfavorable endothelial milieu. This review has shown that biomarkers are associated with unfavorable outcomes after surgery. We hope that more clinicians and researchers will focus their attention to discover if these associations can be of help in the prediction of outcomes. Moreover, changes in dynamic markers that occur at a perioperative phase may, in some cases, have a strong correlation with post-operatory outcome. However, a panel of biomarkers should be identified in order to help the clinician in the assessment of early and late risk of negative outcomes after the intervention. In addition, studies should be carried out on the link between these markers and the identification of the underlying molecular mechanisms, which can become the objective for targeted therapies. In our opinion, translational cardiovascular research needs to fill the gap between molecular mechanisms and a mere biomarker evaluation.

## Conflict of Interest Statement

The authors declare that the research was conducted in the absence of any commercial or financial relationships that could be construed as a potential conflict of interest.
