# Supplementary material for: Biomarkers in Coronary Artery Bypass Surgery: Ready for Prime Time and Outcome Prediction?
Source: Front Cardiovasc Med. 2016 Jan 5;2:39. doi: 10.3389/fcvm.2015.00039 (PMC4700141; doi:10.3389/fcvm.2015.00039)
Supplement: Supplementary file 1 [file Table_1.DOC]

**Supplemental Table 1**

Genetic markers potentially influencing inflammatory response levels and perioperative outcomes after coronary bypass surgery.

|  | **Author** | **Years** | **Protein** | **Polymorphism/**  **genetic mutation** | **Patients** | | **Blood collection timing** | **Biomarker levels** | **Outcome** |
| --- | --- | --- | --- | --- | --- | --- | --- | --- | --- |
| **INFLAMMATION** | **Burzotta**  **et al.**  [18] | 2001 | IL -6 | SNP -174 G>C | CABG | 111 | Preop, 24h, 48h, 72h after surgery and at discharge | IL6-174 GG genotype is associated with higher IL-6 levels in the early days after surgery. | GG genotype is associated with longer ICU and hospital stay. The composite endpoint of postoperative death, myocardial infarction, and stroke were higher in GG carriers (8% vs 2%) but this did not reach statistical significance. |
|  | **Podgoreanu**  **et al.**  *PEGASUS Study*  [31] | 2006 | IL -6 | SNP -572 G>C  (overall, 48 SNPs from 23 genes involved in inflammatory, endothelial activation pathways were studied see below ) | CABG  PMI  No PMI | 434  52  382 | Baseline, 4.5h, 24h and 48h after aortic cross-clamp removal | n.a. | IL6 -572 G>C (OR= 2.47) is an independent predictor of perioperative MI, defined as CKMB≥10 upper limit of normal at 24 hours postop |
|  | **Sanders**  **et al.**  [19] | 2009 | IL -6 | SNPs:  -174 G>C  -572 G>C | CABG  (Caucasian pts.) | 366 | Preop, 6h and 24h after surgery | IL-6 572 C allele is associated with overall higher IL-6 levels after surgery. | Both polymorphisms are not associated with postoperative complications. |
|  | **Wypasek**  **et al.**  [26] | 2010 | IL -6 | SNP -174 G>C | CABG | 179 | Before surgery and at discharge | C allele carriers have increased IL-6 and CRP levels at baseline; moreover, they have increased CRP and a trend toward increased IL-6 levels at discharge (essays on 87 pts). | No association with outcome |
|  | **Brull**  **et al.**  [29] | 2003 | CRP | SNPs:  -717 G>A  +1444 C>T | CABG | 193 | Preop  POD from 1 to 5 days | CRP is higher in +1444TT homozygotes than +144 C-allele especially at 72 h after surgery | n.a. |
|  | **Lobato**  **et al**.  *PEGASUS Study*  [20] | 2011 |  | SNPs:  CRP, IL-1α, IL-1β, IL-6, IL-8, IL-10, IL-1RN, TNFα  (overall, 90 SNPs from 49 genes involved in thrombosis, renin-angiotensin system and endothelial dysfunction were studied; see above and below) | CABG  Discovery cohort  Validation cohort | 1018  930 | n.a. | n.a. | No association between inflammatory (CRP, IL-1α, IL-1β, IL-6, IL-8, IL-10, IL-1RN, TNFα) SNPs and 5-year all-cause mortality. |
|  | **Perry**  **et al.**  [28] | 2009 | CRP | 17 different SNPs | CABG | 604 | Preoperative;  Immediately after the end of surgery;  POD from 1 to 4 day | T allele of rs3091244 associated with higher CRP levels on POD 2 to 3;  C allele of rs1800947 is associated with lower CRP levels on POD 2 to 4. | n.a. |
|  | **Podgoreanu**  **et al.**  *PEGASUS Study*  [31] | 2006 | CRP | SNPs -1846 C>T  (overall, 48 SNPs from 23 genes involved in inflammatory, endothelial activation pathways were studied; see below ) | CABG  PMI  No PMI | 434  52  382 | Baseline, 4.5h, 24h and 48h after aortic cross-clamp removal | n.a. | CRP -1846 C>T is associate with incident PMI, defined as CK-MB≥10 upper limit of normal at 24 hours postop. |
|  | **Wypasek**  **et al.**  [21] | 2012 | CRP, IL6 | SNP –C148T | CABG | 243 | Preop and at discharge | T allele is associated with increased preop CRP levels, and with higher CRP and IL-6 levels at discharge. (essays on 124 pts) | T allele carriers have an increased risk of non-fatal ischemic stroke during postoperative period. Patients with stroke had higher CRP levels than those without this complication. |
|  | **Boehm**  **et al.**  [24] | 2011 | TNF-α | SNPs:  -863 C/A  -308 G/A | CABG | 122 | Preop, 24h, 48h, 72h after surgery and at discharge | TNF-α levels are higher for carriers of -863CC variant preoperatively and up to 6 postoperative hours. -308AA carriers have higher preoperative TNF-α levels, whereas -308GG carriers show lower TNF-α after CPB. | No significant associations between the different genotypes of TNF –α - 863 C/A or -308 G/A with major adverse postoperative events. |

**Abbreviations: SNP**, Single Nucleotide Polymorphism; **IL**, Interleukin; **ICU**, Intensive Care Unit; **CABG**, Coronary Artery Bypass Graft; **MI**, Myocardial Infarction; **Preop**, preoperative; **CRP,** C-reactive Protein; **IL1RN**, Interleukin 1-receptor antagonist; **TNF-α,** Tumor Necrosis Factor α; **Postop,** postoperative; **POD**, Postoperative days; **CK-MB**, Creatine Kinase muscle-brain; **PMI**, Postoperative Myocardial Infarction; **CPB**, Cardiopulmonary Bypass.
