# Supplementary material for: Biomarkers in Coronary Artery Bypass Surgery: Ready for Prime Time and Outcome Prediction?
Source: Front Cardiovasc Med. 2016 Jan 5;2:39. doi: 10.3389/fcvm.2015.00039 (PMC4700141; doi:10.3389/fcvm.2015.00039)
Supplement: Supplementary file 2 [file Table_2.DOC]

**Supplemental Table 2**

Genetic markers potentially influencing hemostasis biomarkers and perioperative outcomes after coronary bypass surgery.

|  | **Author** | **Years** | **Protein** | **Polymorphism/**  **genetic mutation** | **Patients** | | **Blood collection timing** | **Biomarker levels** | **Outcome** |
| --- | --- | --- | --- | --- | --- | --- | --- | --- | --- |
| **HEMOSTATIS** | **Emiroglu**  **et al.**  [22] | 2011 | Factor V Leiden  Prothrombin/factor II | SNPs:  FVL 1691 G/A  PT G20210A | CABG | 220 | n.a. | n.a | No association with perioperative mortality. |
|  | **Lobato**  **et al.**  *PEGASUS*  *Study*  [20] | 2011 |  | 90 SNPs from 49 genes involved in inflammation, renin-angiotensin system and endothelial dysfunction were studied (see above and below) | CABG  Discovery cohort  Validation cohort | 1018  930 | n.a. | n.a. | Two linked common allelic variants of thrombomodulin gene (THBD rs 1042579, HR=2.26, and THBD rs 3176123, HR=3.6) are associated with 5-year all-cause mortality .  No association for TF, TFPI, fibrinogen beta and gamma chains, platelet glycoproteins, p-selectin, and PAI-1 SNPs. |
|  | **Stepien**  **et al.**  [21] | 2011 | E-selectin | SNP S149R | CABG  CPB  OPCAB | 152  134 | Preop and 18-24 h after surgery | S149R allele is associated with increased postop β-thromboglobulin levels (148 essays in total). | n.a. |
|  | **Volzke**  **et al.**  [33] | 2005 | Glycoprotein IIIa  Factor V Leiden | SNPs:  PlA1/PlA2  FVL 1691 G/A | CABG | 247 | n.a. | n.a. | No association of these SNPs with the 2-years all causes of mortality and with cardiac mortality plus need of revascularization during follow-up. There is a trend for reduced incidence of both end-points in glycoprotein IIIa PlA2/PlA2 omozygotes. |
|  | **Welsby**  **et al.**  *PEGASUS substudy*  [23] | 2005 |  | 17 coagulation, fibrinolysis and platelet glycoprotein SNPs plus ACE and MTHFR SNPs | CABG | 759 | n.a | n.a. | 6 SNPs are associated with postop bleeding with each other: GPIaIIa -52C>T and 807C>T, GPIbα 524C>T, TF -603A>G, prothrombin 20210G>A, TFPI -399C>T. |
|  | **Wypasek**  **et al.**  [27] | 2012 | FGB | SNP –C148T | CABG | 124 | Preop and at discharge | 148 T allele carriers is associated with increased fibrinogen levels at baseline. | T allele carriers have an increased risk of perioperative non-fatal stroke. |

**Abbreviations: SNP**, Single Nucleotide Polymorphism; **CABG**, Coronary Artery Bypass Graft; **Preop**, preoperative; **Postop,** postoperative; **PAI-1**, Plasminogen Activator Inhibitor 1; **F5**, Coagulation Factor V; **PT**, Prothrombin; **FVL**, Factor V Leiden; **THBD**, Thrombomodulin;; **CPB**, Cardiopulmonary Bypass, **FGB**, Fibrinogen beta-chain; **TF**, Tissue Factor; **TFPI**, Tissue Factor Pathway Inhibitor; **ACE**, Angiotensin converting enzyme; **MTHFR**, Methylentetrahydrofolate reductase.
