# Supplementary material for: Biomarkers in Coronary Artery Bypass Surgery: Ready for Prime Time and Outcome Prediction?
Source: Front Cardiovasc Med. 2016 Jan 5;2:39. doi: 10.3389/fcvm.2015.00039 (PMC4700141; doi:10.3389/fcvm.2015.00039)
Supplement: Supplementary file 3 [file Table_3.DOC]

**Supplemental Table 3**

Genetic markers potentially influencing renin angiotensin system and perioperative outcomes after coronary bypass surgery.

|  | **Author** | **Years** | **Protein** | **Polymorphism/**  **genetic mutation** | **Patients** | | **Blood collection timing** | **Biomarker levels** | **Outcome** |
| --- | --- | --- | --- | --- | --- | --- | --- | --- | --- |
| **RENIN-ANGIOTENSIN** | **Dayi**  **et al.**  [34] | 2005 | ACE | SNP ACE I/D | CABG | 87 | n.a. | n.a. | ACE D/D genotype is associated with reduced saphenous graft patency. |
|  | **Emiroglu**  **et al.**  [22] | 2011 | ACE | SNP ACE I/D | CABG | 220 | n.a. | n.a. | No association with perioperative mortality. |
|  | **Lobato**  **et al.**  *PEGASUS Study*  [20] | 2011 |  | SNPs:  ACE, Angiotensin  (overall 90 SNPs from 49 genes involved in inflammation, thrombosis, and endothelial dysfunction were studied; see above and below) | CABG  Discovery cohort  Validation cohort | 1018  930 | n.a. | n.a. | No association between ACE and angiotensin SNPs and 5-year all-cause mortality. |
|  | **Welsby**  **et al.**  *PEGASUS substudy*  [23] | 2005 | ACE | SNP ACE Insertion/Deletion (I/D) | CABG | 759 | n.a. | n.a. | Independently associated with postop bleeding |
|  | **Volzke**  **et al.**  [35] | 2002 | ACE | SNP ACE I/D | CABG | 249 | n.a. | n.a. | ACE D/D genotype is associated to both 2-years to mortality (OR=2.5) and to cardiac mortality plus need of revascularization (OR=3.1). |

**Abbreviations: SNP**, Single Nucleotide Polymorphism; **CABG**, Coronary Artery Bypass Graft; **Postop,** postoperative; **ACE**, Angiotensin converting enzyme.
