# Supplementary material for: Biomarkers in Coronary Artery Bypass Surgery: Ready for Prime Time and Outcome Prediction?
Source: Front Cardiovasc Med. 2016 Jan 5;2:39. doi: 10.3389/fcvm.2015.00039 (PMC4700141; doi:10.3389/fcvm.2015.00039)
Supplement: Supplementary file 4 [file Table_4.DOC]

**Supplemental Table 4**

Genetic markers potentially influencing endothelial protein levels and perioperative outcomes after coronary bypass surgery.

|  | **Author** | **Years** | **Protein** | **Polymorphism/**  **genetic mutation** | **Patients** | | **Blood collection timing** | **Biomarker levels** | **Outcome** |
| --- | --- | --- | --- | --- | --- | --- | --- | --- | --- |
| **ENDOTHELIAL FUNCTION** | **Liakopoulos**  **et al.**  [36] | 2006 | eNOS | SNPs:  894G/T  -786C/T  (difference of vascular reactivity) | CABG | 105 | Systemic hemodynamics, cardiac index (CI), systemic and pulmonary vascular resistance indices (SVRI, PVRI) and catecholamine support at baseline and 1 h, 4 h, 10 h and 24 h after surgery | 894G/T and -786C/T genetic polymorphisms of the eNOS gene do not influence early perioperative hemodynamics after cardiac surgery | No association between eNOS SNPs and hospital mortality, ICU and hospital stay, ventilation time, renal failure and MI. |
|  | **Lobato**  **et al.**  *PEGASUS Study*  [20] | 2011 |  | SNPs:  E-selectin, ICAM1, NOS3, PECAM1, VCAM1  (overall 90 SNPs from 49 genes involved in inflammation, thrombosis and renin-angiotensin system were studied; see above and below) | CABG  Discovery cohort  Validation cohort | 1018  930 | n.a. | n.a. | No association between E-selectin, ICAM1, NOS3, PECAM1 VCAM1 SNPs and 5-year all-cause mortality |
|  | **Podgoreanu**  **et al.**  *PEGASUS Study*  [31] | 2006 | ICAM1  SELE | SNPs:  Lys469Glu  -98G>T  (overall 48 SNPs from 23 genes involved in inflammatory pathways were studied; see above). | CABG  PMI  No PMI | 434 52  382 | Baseline, 4.5h, 24h and 48h after aortic cross-clamp removal. | n.a. | ICAM1 Lys469Glu (OR= 1.88), SELE -98G>T (OR=0.16) are independent predictors of perioperative MI, defined as CKMB≥10 upper limit of normal at 24 hours postop |
|  | **Ragia**  **et al.**  [37] | 2010 | eNOS | SNPs:  894 G>T  -786 C>T | CABG  Controls | 154  155 | n.a | n.a. | No association between genotypes and the occurrence of severe coronary disease requiring intervention. |
|  | **Stepien**  **et al.**  [21] | 2011 | E-selectin | SNP S149R | CABG | 152 | n.a. | n.a. | S149R allele is associated with increased adverse events (MI, stroke, PE) early after surgery (RR=2.03). |

**Abbreviations: SNP**, Single Nucleotide Polymorphism; **ICU**, Intensive Care Unit; **CABG**, Coronary Artery Bypass Graft; **MI**, Myocardial Infarction; **Preop**, preoperative; **Postop,** postoperative; **CK-MB**, Creatine Kinase muscle-brain; **PMI**, Postoperative Myocardial Infarction; **NOS3/eNOS**, Endothelial nitric oxide synthase; **ICAM1**, Intercellular adhesion molecule-1; **PECAM1**, Platelet-endothelial cell adhesion molecule-1; **VCAM1,** vascular cell adhesion molecule-1, **SELE**, E-selectin, **PE**, pulmonary embolism.
