# Supplementary material for: Biomarkers in Coronary Artery Bypass Surgery: Ready for Prime Time and Outcome Prediction?
Source: Front Cardiovasc Med. 2016 Jan 5;2:39. doi: 10.3389/fcvm.2015.00039 (PMC4700141; doi:10.3389/fcvm.2015.00039)
Supplement: Supplementary file 5 [file Table_5.DOC]

**Supplemental Table 5**

Genetic markers potentially influencing other pathways levels and perioperative outcomes after coronary bypass surgery.

|  | **Author** | **Years** | **Protein** | **Polymorphism/**  **genetic mutation** | **Patients** | | **Blood collection timing** | **Biomarker levels** | **Outcome** |
| --- | --- | --- | --- | --- | --- | --- | --- | --- | --- |
|  | **Liu**  **et al.**  *CABG Genomics study*  [24] | 2010 |  | 61 SNPs on 9p21 | CABG | 846 | CK-MB, B-type natriuretic peptide, cTnI (preop, protamine, 1,2,3,4,5 days postop). cTnI > 9.13 µg/L on postop day 1 as indicator of perioperative MI. | SNPs rs 10116277, rs 6475606, rs 2383207 are associated with increased postoperative cTnI levels even after accounting for clinical covariates and multiple comparisons. | SNPs rs 10116277 (OR=1.79), rs 6475606 (OR=1.79), rs 2383207 (OR=1.71) are associated with perioperative MI. |
|  | **Muehlschlegel**  **et al.**  *CABG Genomics study*  [25] | 2010 |  | SNP rs 10116277 on 9p21 | CABG | 846 | n.a. | n.a. | The homozygote minor allele of rs 10116277 is associated with significantly increased risk of all-cause mortality at 5 years (HR=1.7) |

**Abbreviations: SNP**, Single Nucleotide Polymorphism; **CABG**, Coronary Artery Bypass Graft; **MI**, Myocardial Infarction; **Preop**, preoperative; **Postop,** postoperative; **CK-MB**, Creatine Kinase muscle-brain; **cTnI**, Cardiac Troponin I; **AR,** Adrenergic Receptor
